# Implantation Outcome-Specific Associations Between Stromal Senescence and the Endometrial Microbiota

**DOI:** 10.3390/microorganisms14091868

**Published:** 2026-08-22

**Authors:** Dimitar Parvanov, Margarita Ruseva, Rumiana Ganeva, Teodora Tihomirova, Maria Handzhiyska, Stela Chapanova, Jinahn Safir, Sofia Koristashevskaya, Ivan Pavlov, Dimitar Metodiev, Blaga Rukova, Georgi Stamenov, Savina Hadjidekova

**Affiliations:** 1 Department of Research and Development, Nadezhda Women’s Health Hospital, 1330 Sofia, Bulgaria; margarita.ruseva@gmail.com (M.R.); rum.ganeva@gmail.com (R.G.); mariavh@abv.bg (M.H.); jinahn.safir@gmail.com (J.S.); koristashevskaya.s@gmail.com (S.K.); ivanypavlov@gmail.com (I.P.); 2 Department of Obstetrics and Gynecology, Nadezhda Women’s Health Hospital, 1330 Sofia, Bulgaria; t.tihomirova@mail.bg (T.T.); stelachapanova@abv.bg (S.C.); g.stamenov1@abv.bg (G.S.); 3 Department of Clinical Pathology, Nadezhda Women’s Health Hospital, 1330 Sofia, Bulgaria; dr.dmetodiev@yahoo.com; 4 Department of Medical Genetics, Nadezhda Women’s Health Hospital, 1330 Sofia, Bulgaria; blagarukova@yahoo.com (B.R.); svhadjidekova@medfac.mu-sofia.bg (S.H.); 5 Department of Medical Genetics, Medical University of Sofia, 1431 Sofia, Bulgaria

**Keywords:** endometrial senescence, stromal senescence, endometrial microbiota, *Lactobacillus*, *Delftia*, implantation window, endometrial receptivity, host–microbiota interactions

## Abstract

Endometrial senescence and the endometrial microbiota have both been implicated in the regulation of endometrial receptivity, yet their relationship remains poorly understood. The aim of this study was to investigate associations between p16-positive endometrial cells and microbiota composition during the implantation window and to determine whether these relationships differ according to implantation outcome. Endometrial senescence was assessed by p16 immunohistochemistry and digital image analysis, whereas microbial composition was characterized by 16S rRNA gene sequencing in endometrial biopsies collected from 68 women prior to undergoing transfer of a single euploid embryo, which was performed within six months of biopsy under the same hormonal preparation protocol. No significant differences in luminal epithelial or stromal p16 abundance were observed according to subsequent implantation outcome. Although senescence was not directly associated with implantation success, stromal p16 expression demonstrated multiple associations with the endometrial microbiota. Increased stromal p16-positivity was associated with lower relative abundance of *Lactobacillus* and higher abundance of *Delftia*. Notably, in exploratory subgroup analyses more pronounced associations were observed in women who achieved pregnancy, including a negative correlation between stromal p16 expression and *Lactobacillus* abundance (ρ = −0.48, *p* = 0.005) and positive correlation with *Delftia* abundance (ρ = 0.42, *p* = 0.016). Species-level analyses revealed implantation outcome-specific associations involving *Lactobacillus iners* and *Limosilactobacillus vaginalis*. In addition, stromal p16 expression was associated with differences in microbial co-occurrence networks, indicating broader effects on microbial community organization. Together, these findings suggest that stromal, but not luminal, senescence is closely linked to endometrial microbiota composition and microbial community organization during the window of implantation. The observation that the strongest senescence–microbiota associations occurred in women who subsequently achieved successful implantation supports the hypothesis that coordinated senescence–microbiota relationships may represent a previously underrecognized feature of the receptive endometrial microenvironment.

## 1. Introduction

Successful embryo implantation depends on the establishment of a receptive endometrial environment capable of supporting embryo attachment, invasion, and early maternal–fetal communication. Endometrial receptivity is a dynamic process regulated by hormonal signaling, immune cell activity, tissue remodeling, and local inflammatory responses, which together create the conditions required for successful implantation and pregnancy maintenance [1,2,3,4].

The introduction of next-generation sequencing (NGS) technologies has challenged the traditional concept of the sterile uterine cavity and has led to the identification of distinct microbial communities within the endometrium [5,6]. Several studies have reported associations between endometrial microbiota composition and reproductive outcomes, particularly in women undergoing assisted reproductive treatment. Particular attention has been directed toward *Lactobacillus* species, which frequently dominate the reproductive tract microbiota and have been associated with favorable implantation and pregnancy outcomes [5,7,8]. Conversely, reduced *Lactobacillus* abundance and increased microbial diversity have often been interpreted as indicators of dysbiosis and impaired reproductive function [5,8,9]. More recently, accumulating evidence suggests that the endometrial microbiota participates in bidirectional interactions with the local immune microenvironment, potentially influencing immune cell recruitment, inflammatory signaling, maternal immune tolerance, and endometrial receptivity during the implantation window [10,11,12].

Although numerous studies support a biological role for the endometrial microbiota, its composition, origin, and clinical significance remain subjects of ongoing debate. Because the endometrium represents a low-biomass environment, bacterial DNA detected by sequencing may be influenced by contamination introduced during sample collection, DNA extraction, library preparation, or sequencing reagents [13,14]. Consequently, several recent reviews have emphasized the importance of rigorous contamination control, the inclusion of appropriate negative controls, standardized sampling procedures, and cautious interpretation of sequencing results in studies investigating the endometrial microbiota [15,16]. Nevertheless, studies employing rigorous contamination control strategies continue to support the presence of distinct bacterial communities within the endometrium [17,18]. These observations further suggest that the endometrial microbiota may contribute to endometrial physiology and reproductive function, although its precise biological role remains to be fully elucidated [5,6,7].

In parallel with advances in microbiota research, cellular senescence has emerged as an important regulator of tissue homeostasis, regeneration, aging, and inflammation [19,20]. Senescent cells undergo stable cell-cycle arrest while remaining metabolically active and producing a broad spectrum of bioactive molecules collectively referred to as the senescence-associated secretory phenotype (SASP) [19,21]. The endometrium represents a unique tissue in which controlled senescence appears to play important physiological roles. Cyclic tissue remodeling, decidualization, embryo implantation, and immune regulation have all been linked to transient senescent cell populations within the endometrium [22,23,24]. Collectively, these observations have shifted the current view of endometrial senescence from a simple marker of tissue aging toward a dynamic biological process that contributes to endometrial homeostasis and reproductive function. At the same time, accumulating evidence indicates that dysregulated or persistent senescence may impair endometrial receptivity and contribute to reproductive disorders [19,20,21,22,23,24]. Consistent with this emerging concept, our previous studies have demonstrated that endometrial senescent cells are closely associated with local immune cell populations, plasma cell infiltration, and the spatial organization of the endometrial immune microenvironment during the implantation window [25,26,27].

Beyond the endometrium, growing evidence from multiple biological systems suggests that interactions between cellular senescence and resident microbial communities represent a conserved biological phenomenon [19,28]. In the intestine, age-related microbial dysbiosis has been linked to chronic low-grade inflammation, oxidative stress, and the accumulation of senescent cells, whereas depletion or modulation of the gut microbiota has been associated with attenuation of several hallmarks of age-associated inflammation [28,29]. Conversely, senescent cells actively remodel their surrounding microenvironment through the senescence-associated secretory phenotype (SASP), altering epithelial barrier integrity, immune responses, extracellular matrix remodeling, and local metabolic conditions that may influence microbial community composition [19,21,30]. Similar reciprocal interactions between microbial dysbiosis and cellular senescence have been described across multiple organs, including the oral cavity, lung, skin and gastrointestinal tract, suggesting that microbiota–senescence interactions may represent a conserved mechanism regulating tissue homeostasis and chronic inflammation [28,31,32].

Collectively, these observations suggest that host cellular senescence and microbial communities may influence each other bidirectionally. Microorganisms can induce senescence through chronic inflammatory stimulation, oxidative stress, and microbial metabolites, whereas senescent cells may alter local ecological conditions through SASP-mediated effects on immune responses and tissue homeostasis [19,33,34]. Such interactions could be particularly relevant in the endometrium, where both microbial composition and cellular senescence are increasingly recognized as important determinants of local tissue function. However, whether specific microbial taxa are associated with endometrial senescence and whether these relationships differ according to implantation outcome remain unknown.

Although endometrial senescence and the endometrial microbiota have each been independently associated with implantation and reproductive outcome, whether these biological processes are interconnected within the receptive endometrium and whether these relationships differ according to subsequent implantation outcome remain unknown. Therefore, we investigated associations between stromal and luminal p16-positive endometrial cells and endometrial microbial composition in women undergoing transfer of a single euploid embryo. In addition, we evaluated whether stromal senescence was associated with alterations in microbial community organization using co-occurrence network analysis.

## 2. Materials and Methods

### 2.1. Study Population and Sample Collection

This prospective observational study was conducted at Nadezhda Women’s Health Hospital (Sofia, Bulgaria). Endometrial biopsies were obtained on progesterone day 5 (P + 5), corresponding to the window of implantation in hormone replacement treatment (HRT) cycles, from women undergoing IVF treatment between March 2022 and February 2025. For all patients, the subsequent frozen embryo transfer was performed using the same standardized HRT protocol, with embryo transfer likewise scheduled on progesterone day 5, thereby reproducing the hormonal conditions under which the biopsy had been obtained.

**Figure 1:**
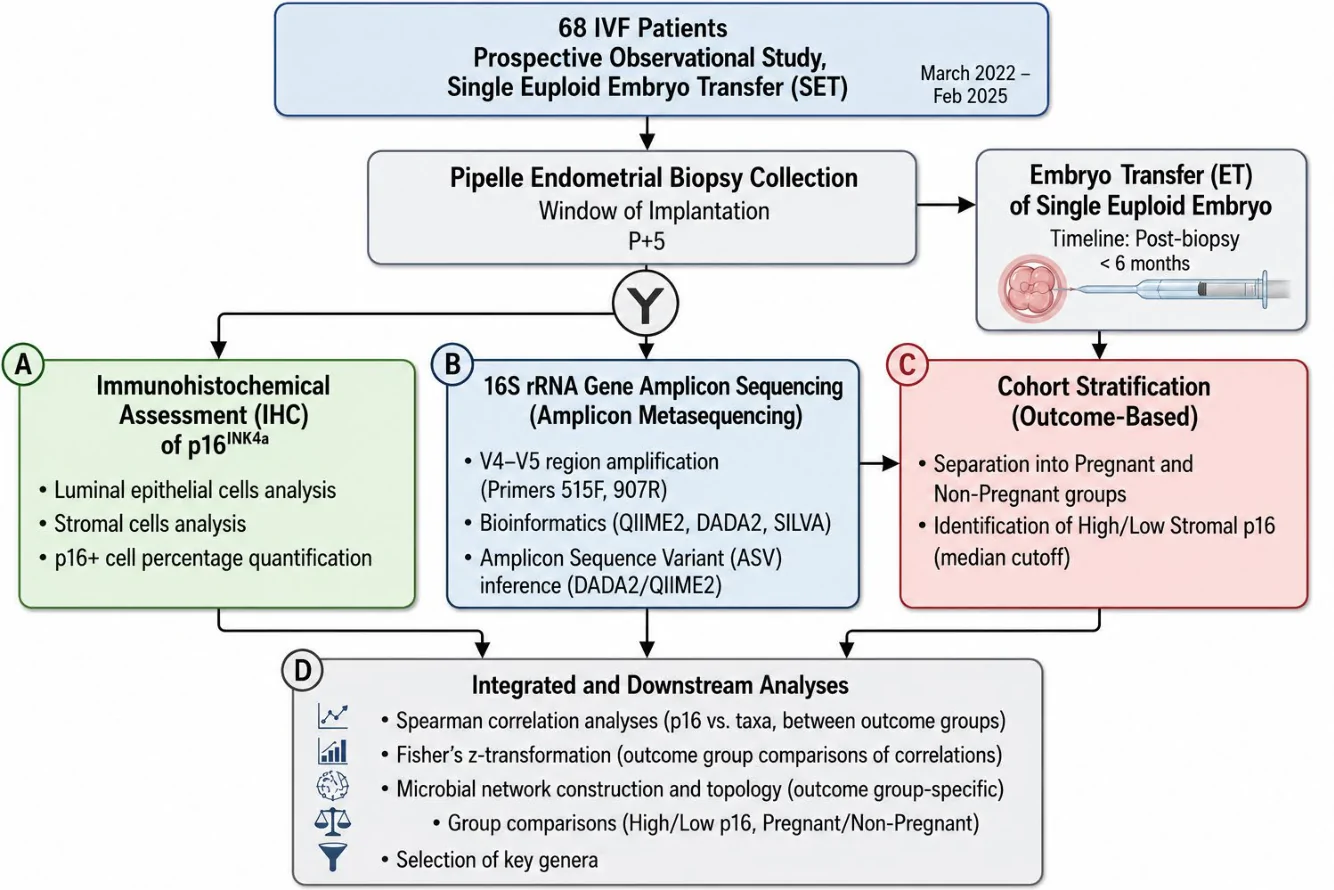
Study design and analytical workflow. The study included 68 women undergoing single euploid embryo transfer. For subgroup analyses, patients were stratified into low- and high-stromal p16 groups using the cohort median stromal p16 percentage as the predefined cutoff. (A) Immunohistochemical assessment of p16INK4a expression in luminal epithelial and stromal cells; (B) 16S rRNA gene amplicon sequencing and microbiota profiling; (C) outcome-based cohort stratification and classification according to stromal p16 expression; (D) integrated and down-stream statistical analyses, including correlation analyses, microbial network analysis, group comparisons, and selection of key genera.

A total of 68 women were included in the present analysis (Figure 1). All participants underwent endometrial biopsy followed by microbiota profiling and quantitative assessment of p16-positive endometrial cells. The transfer of a single euploid embryo as confirmed by preimplantation genetic testing for aneuploidy (PGT-A) was performed within 6 months after the biopsy. Implantation outcome was evaluated by serum human chorionic gonadotropin (hCG) test 14 days after embryo transfer.

Endometrial tissue was obtained using a Pipelle catheter (Laboratoire CCD, Paris, France) under sterile conditions. Following collection, samples were divided for microbiota profiling and histological evaluation. Only biopsy specimens providing sufficient endometrial tissue for both microbiota profiling and histopathological evaluation were included in the study. Biopsies yielding insufficient tissue for reliable histological assessment, including inadequate representation of the luminal epithelium when required for compartment-specific analyses, were not included in the analytical cohort. All endometrial biopsies underwent routine histopathological evaluation. Chronic endometritis was assessed by immunohistochemical detection of CD138-positive plasma cells as part of the pathological assessment. No patients fulfilling the diagnostic criteria for chronic endometritis were included in the final study cohort. Women with known endometrial pathology, including endometriosis, adenomyosis, endometrial polyps, endometrial hyperplasia, endometrial carcinoma, histologically confirmed chronic endometritis, or evidence of active genital tract infection, were excluded. Endometrial biopsies were obtained before embryo transfer and before initiation of any additional implantation-directed or microbiota-modulating treatment.

All participants provided written informed consent before enrollment. The study protocol was approved by the Institutional Review Board of Nadezhda Women’s Health Hospital (Approval No. 56/29.12.2020) and was conducted in accordance with the Declaration of Helsinki.

### 2.2. Immunohistochemical Assessment of Endometrial Senescence

Endometrial senescence was evaluated by immunohistochemical detection of *p16INK4a*, a widely recognized marker of cellular senescence [26,35]. Immunohistochemical staining was performed using the Novolink Polymer Detection System (Leica Biosystems, Newcastle, UK) according to the manufacturer’s instructions. Cellular senescence was assessed using a mouse monoclonal antibody against human *p16INK4a* (MAD-000690QD-7, Master Diagnostica, Madrid, Spain). Positive and negative controls were included in each staining run to verify antibody performance and staining specificity.

Formalin-fixed paraffin-embedded endometrial tissue sections were stained and representative microscopic fields were imaged using an Olympus CKX41 microscope equipped with an EP50 digital camera (Olympus Europa SE & Co. KG, Hamburg, Germany) at 40× magnification. Senescent cells were identified based on positive nuclear and/or nuclear-cytoplasmic p16 immunoreactivity. Separate analyses were performed for luminal epithelial cells and stromal cells.

### 2.3. Digital Image Analysis

Digital image analysis was performed using HALO Image Analysis software (version 3.4, Indica Labs, Corrales, NM, USA). Representative endometrial images were evaluated separately within the luminal epithelial and stromal compartments.

For luminal epithelial analysis, p16 expression was quantified as the percentage of p16-positive epithelial cells. For stromal analysis, positive cells were quantified relative to the total number of stromal nuclei, excluding glandular epithelium, vascular structures, and luminal epithelial cells. Stromal senescence was expressed as the percentage of p16-positive stromal cells.

All images were analyzed using identical image acquisition and HALO analysis parameters to ensure consistency across samples.

Before quantitative analysis, the HALO image analysis algorithm was optimized using representative endometrial sections to ensure appropriate tissue segmentation and identification of p16-positive cells. The same predefined analysis parameters were then applied uniformly to all study samples. All segmented images and quantitative outputs were visually reviewed to confirm accurate compartment delineation and cell classification.

### 2.4. Amplicon Metasequencing

Endometrial microbial communities were characterized by 16S rRNA gene amplicon metasequencing.

Genomic DNA was isolated from endometrial biopsy specimens using the Quick-DNA Fecal/Soil Microbe Microprep Kit (D6010, Zymo Research, Irvine, CA, USA) according to the manufacturer’s protocol. DNA quantity and purity were determined using a NanoDrop™ 2000c spectrophotometer (Thermo Fisher Scientific, Waltham, MA, USA).

The V4–V5 region of the bacterial 16S rRNA gene was amplified using universal primers 515F and 907R [36,37]. Following amplification, PCR products were confirmed by agarose gel electrophoresis, purified, quantified, and pooled in equimolar amounts for library preparation.

Sequencing libraries were generated according to standard Illumina workflows and sequenced by Novogene Co., Ltd. (Cambridge, UK) on the Illumina HiSeq platform (Illumina, Inc., San Diego, CA, USA) using 2 × 250 bp paired-end reads, with a target sequencing depth of approximately 30,000 reads per sample.

Because endometrial tissue represents a low-biomass microbial environment, contamination control was incorporated throughout sample collection and laboratory processing. Endometrial biopsies were collected under sterile conditions using sterile Pipelle catheters, and sterile instruments were used throughout sample handling.

To monitor potential background contamination, three types of negative controls were included: (i) sterile distilled water, (ii) sterile phosphate-buffered saline (PBS) (VWR, Radnor, PA, USA) used for biopsy storage, and (iii) PBS processed through the complete DNA extraction, PCR amplification, library preparation, and sequencing workflow. All controls were processed and sequenced alongside the clinical specimens and were subsequently incorporated into the ASV-level contamination assessment described below.

### 2.5. Bioinformatics Processing

Sequence data were analyzed using the QIIME2 pipeline (version 2022.2) [38]. Paired-end reads were quality filtered, denoised, merged, and screened for chimeric sequences using the DADA2 algorithm, which infers amplicon sequence variants (ASVs) rather than clustering reads into operational taxonomic units (OTUs) [39].

Taxonomic classification of ASVs was performed using the classify-sklearn plugin in QIIME2 (version 2022.2) with a pre-trained Naïve Bayes classifier against the SILVA SSU rRNA reference database (release 138.2) [40,41]. Taxa were assigned across multiple hierarchical levels, including phylum, class, order, family, genus, and species. Species-level taxonomic assignments were based on the ASV sequences generated using the q2-DADA2 plugin (version 2022.2.0) and the confidence scores obtained using the QIIME2 classify-sklearn classifier trained on the SILVA 138.2 reference database. No additional phylogenetic reconstruction was performed.

Potential background contaminants were evaluated at the ASV level using the prevalence-based approach implemented in the R package decontam (version 1.26.0), with the classification threshold set at 0.10. The three negative controls were analysed together with the endometrial samples, and ASVs with prevalence patterns exceeding this threshold were flagged as candidate contaminants. The resulting candidate ASVs were then linked to their genus-level taxonomic assignments and compared with the taxa retained for the abundance, correlation, and network analyses. Because contaminant classification was performed at the individual ASV level, the detection of a candidate feature assigned to a particular genus was not interpreted as evidence that all ASVs belonging to that genus were contaminants. The screening results were therefore used to assess the robustness of the principal findings rather than to remove entire genera indiscriminately. A genus-level summary of the identified candidate contaminant ASVs is provided in Table A1.

To minimize biases related to unequal sequencing depth, ASV tables were normalized to the sample with the lowest read count prior to statistical analyses.

For genus-level association analyses, taxa were filtered using combined abundance and prevalence criteria, retaining genera with a mean relative abundance of at least 0.1% across the cohort and detection in at least 10% of samples. For exploratory co-occurrence network analyses, a broader prevalence-based filter was applied to preserve sufficient community complexity while excluding sparsely detected taxa. Networks were therefore constructed using bacterial genera detected in at least 10% of study samples.

Species-level analyses were restricted to taxa detected in at least 20% of samples in order to reduce data sparsity and improve the robustness of correlation-based analyses.

### 2.6. Statistical Analysis

Statistical analyses were performed using R version 4.4.2 (R Foundation for Statistical Computing, Vienna, Austria) and IBM SPSS Statistics version 21.0 (IBM Corp., Armonk, NY, USA).

Continuous variables were assessed for normality and are presented as mean ± standard deviation (SD) or median with interquartile range (IQR), as appropriate. Comparisons between groups were performed using Student’s *t*-test or Mann–Whitney U test for continuous variables and Fisher’s exact test or chi-square test for categorical variables.

Associations between the endometrial senescence marker p16 and the genera retained after abundance- and prevalence-based filtering were evaluated using Spearman rank correlation analysis.

To investigate implantation outcome-specific associations, correlation analyses were performed separately in pregnant and non-pregnant patients. Differences between correlation coefficients were assessed using Fisher’s z-transformation.

For exploratory analyses, patients were stratified into low and high stromal p16 groups using the cohort median stromal p16 value as the cutoff. Differences in microbial abundance between groups were evaluated using the Mann–Whitney U test. The cohort median was selected a priori as a distribution-based threshold to generate similarly sized comparison groups for exploratory analyses and did not represent a biologically or clinically validated diagnostic cutoff.

Microbial co-occurrence networks were constructed using genus-level abundance data. Genus-level co-occurrence networks were constructed separately for the low- and high-stromal p16 groups using the broader prevalence-based set of bacterial genera detected in at least 10% of study samples, as described above. Network edges were defined by significant pairwise Spearman correlations (*p* < 0.05). Network topology was characterized using the number of nodes, number of edges, network density, clustering coefficient, average degree, average interaction strength, and node degree centrality. The network analyses were intended as an exploratory characterization of microbial community organization rather than a formal statistical comparison of network topology. Accordingly, network-derived metrics were interpreted descriptively and no formal statistical comparison of network topology was performed.

Multiple testing correction using the Benjamini–Hochberg false discovery rate (FDR) procedure was applied to analyses involving simultaneous testing of multiple taxa (e.g., differential correlation analyses). Exploratory analyses based on predefined hypotheses or descriptive network analyses were interpreted without formal FDR adjustment. All tests were two-sided, and *p*-values < 0.05 were considered statistically significant.

## 3. Results

### 3.1. Patients

The final study cohort consisted of 68 women, including 32 pregnant and 36 non-pregnant patients. No significant differences were observed between the groups regarding age, BMI, reproductive history, endometrial thickness, interval between biopsy and embryo transfer, or infertility diagnosis (all *p* > 0.05) (Table 1).

### 3.2. p-16 Positive Senescent Cells and Implantation Outcome

**Figure 2:**
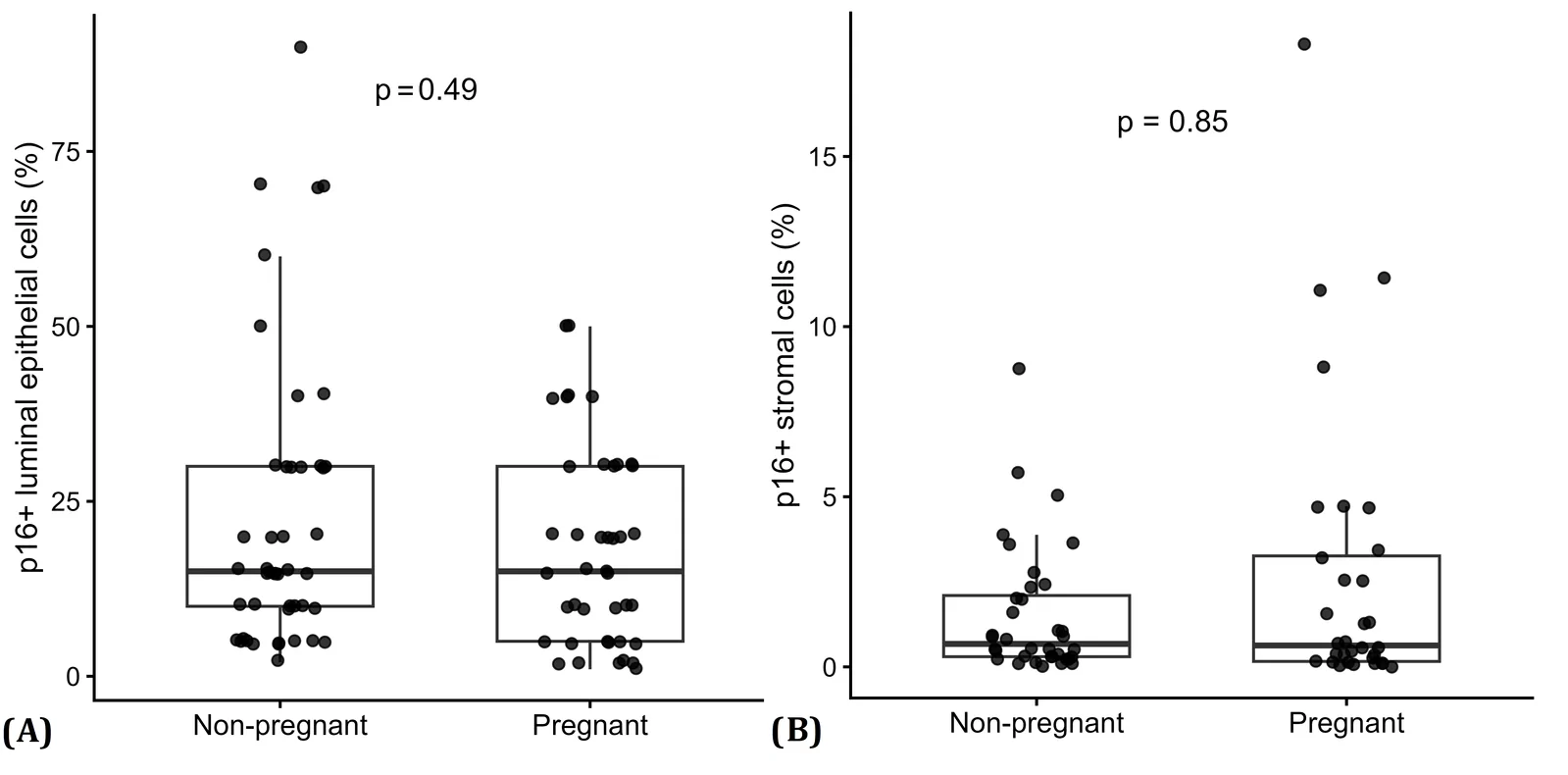
Endometrial senescent cell abundance according to implantation outcome. (**A**) Percentage of p16-positive luminal epithelial cells in women with successful and unsuccessful implantation. (**B**) Percentage of p16-positive stromal cells in women with successful and unsuccessful implantation. Boxes represent the interquartile range (IQR), horizontal lines indicate the median, whiskers represent 1.5 × IQR, and dots correspond to individual patients. Group comparisons were performed using the Mann–Whitney U test.

The abundance of p16-positive cells was compared between women who achieved implantation and those who did not. No significant differences were observed in the proportion of p16-positive luminal epithelial cells between the Pregnant and Non-pregnant groups (*p* = 0.49; Figure 2A). Similarly, stromal p16-positive cell abundance did not differ significantly according to implantation outcome (*p* = 0.85; Figure 2B). These findings indicate that p16-positive cell abundance was not directly associated with implantation success in this cohort.

### 3.3. Prevalence-Based Assessment of Potential Background Contamination

Prevalence-based screening of the negative controls identified 14 candidate contaminant ASVs distributed across 11 bacterial genera (Table A1). The number of candidate contaminant ASVs assigned to an individual genus ranged from one to three. For most of the identified genera, candidate contaminant ASVs represented only a subset of the total ASVs assigned to that genus, although the proportion varied among taxa. Importantly, no candidate contaminant ASVs were assigned to *Lactobacillus* and *Delftia*, which contributed to the principal genus-level associations reported in the present study. The complete genus-level summary, including the total number of ASVs and the number classified as candidate contaminants, is provided in Table A1.

### 3.4. Association Between Stromal p16 Senescence and the Endometrial Microbiota

Application of the predefined abundance- and prevalence-based filtering criteria retained six annotated genera for genus-level analyses (*Lactobacillus*, *Cutibacterium*, *Delftia*, *Staphylococcus*, *Litchfieldia*, and *Allobaculum*). Individual endometrial microbiota profiles showed substantial inter-individual variability in the relative abundance of these taxa (Supplementary Table S1).

**Figure 3:**
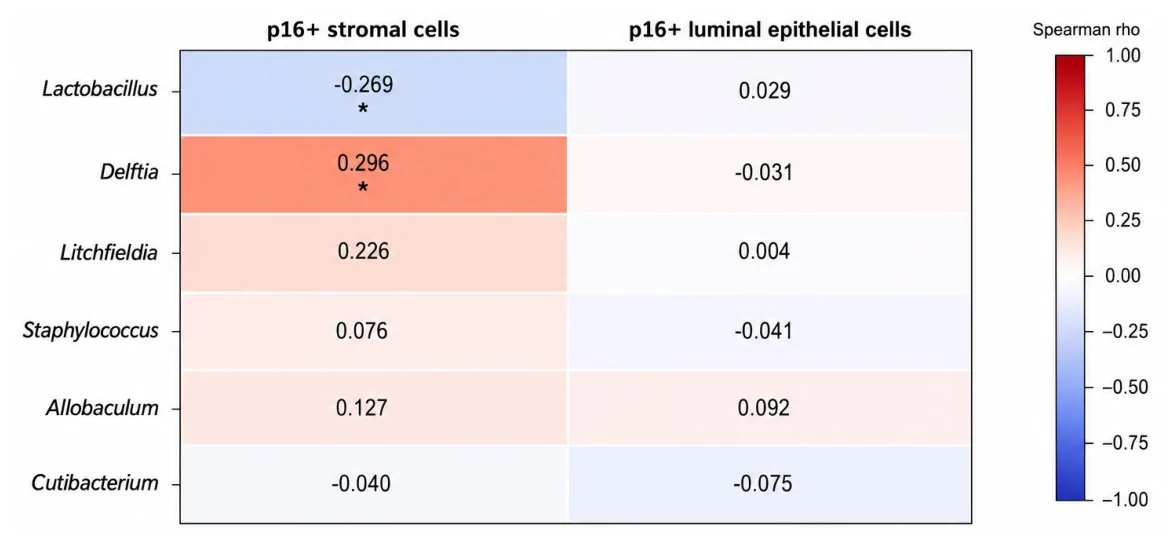
Correlations between endometrial p16-positive cells and bacterial genera retained after abundance- and prevalence-based filtering. Heatmap showing Spearman correlation coefficients between bacterial relative abundance and the proportions of p16-positive luminal epithelial and stromal cells. Color intensity reflects the strength and direction of the correlation. Values within cells indicate Spearman correlation coefficients (ρ). Asterisks indicate nominal correlations (*p* < 0.05).

Correlation analysis between stromal p16-positive cells and the six retained genera identified nominal associations with two genera (Figure 3). Stromal p16 positivity was negatively correlated with *Lactobacillus* abundance (Spearman ρ = −0.269, *p* = 0.027, q = 0.079) and positively correlated with *Delftia* abundance (ρ = 0.296, *p* = 0.014, q = 0.079). However, neither association remained statistically significant after Benjamini–Hochberg FDR correction. No significant associations were observed between luminal epithelial p16-positive cells and the analyzed genera after correction for multiple testing.

### 3.5. Implantation Outcome-Stratified Associations Between Stromal p16 Expression and Bacterial Taxa

**Figure 4:**
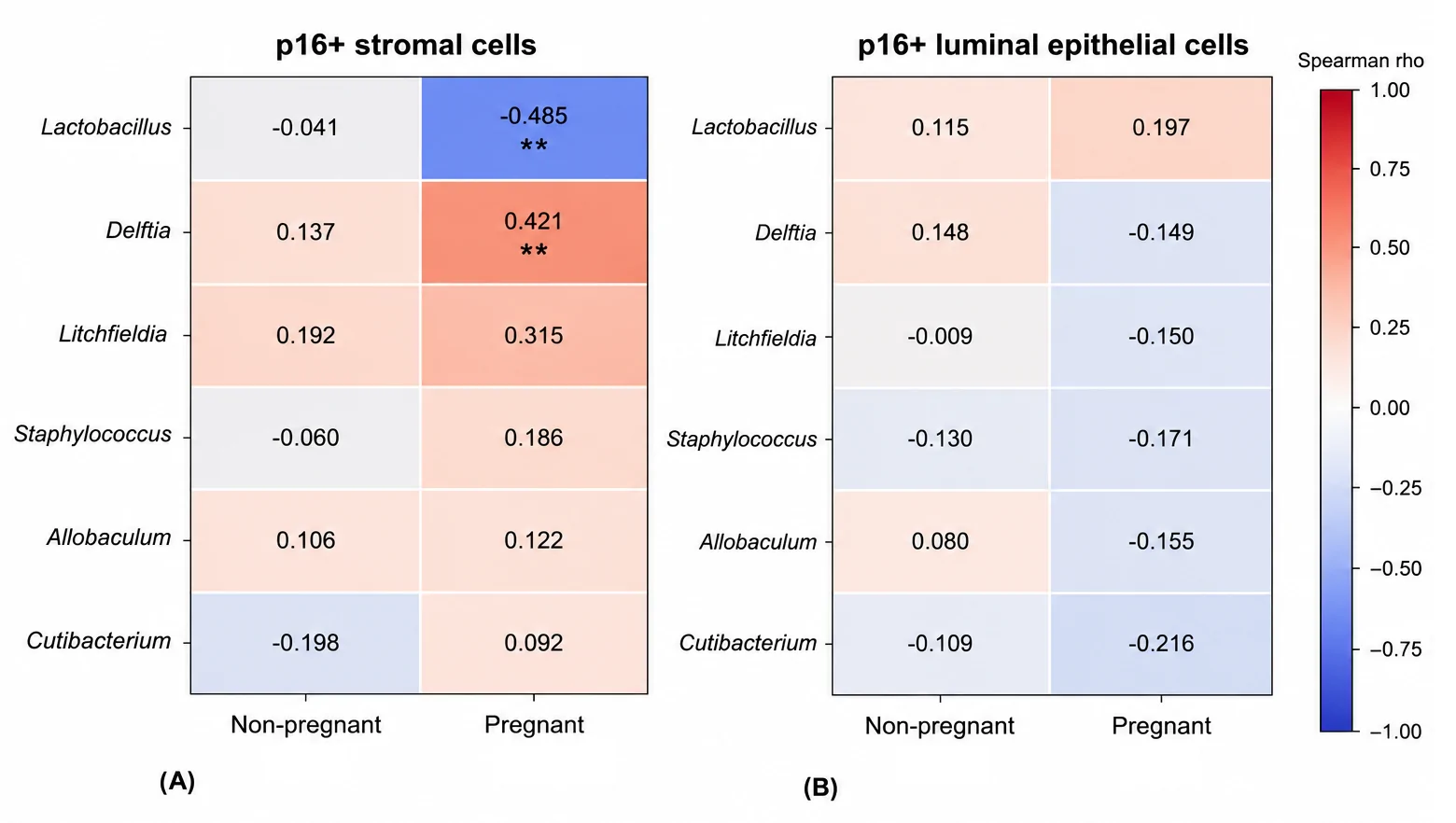
Implantation outcome-stratified associations between endometrial p16 expression and bacterial genera retained after abundance- and prevalence-based filtering. (**A**) Correlations between p16-positive stromal cells and bacterial genera according to implantation outcome; (**B**) correlations between p16-positive luminal epithelial cells and bacterial genera according to implantation outcome. Heatmaps display Spearman correlation coefficients separately for non-pregnant and pregnant patients. Values within cells indicate Spearman correlation coefficients (ρ), and color intensity reflects the strength and direction of the correlation. Asterisks ** indicate correlations remaining significant after Benjamini–Hochberg FDR correction (q < 0.05).

**Figure 5:**
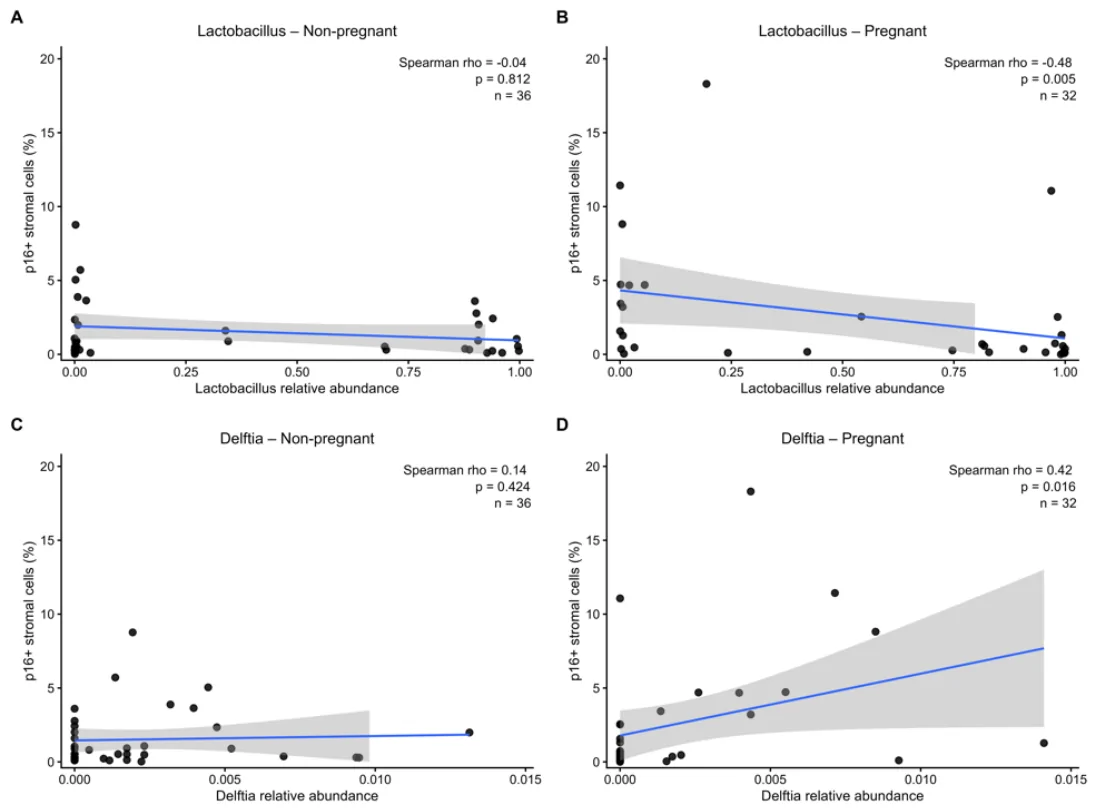
Correlations between p16+ stromal cells and the relative abundance of *Lactobacillus* and *Delftia* according to implantation outcome. Scatterplots showing associations between the percentage of p16-positive stromal cells and the relative abundance of *Lactobacillus* (**A**,**B**) and *Delftia* (**C**,**D**) in non-pregnant and pregnant patients. Spearman correlation coefficients (ρ), *p*-values, and sample sizes are shown within each panel. Blue lines represent fitted regression lines, and gray shaded areas indicate the corresponding 95% confidence intervals.

To determine whether these relationships differed according to implantation outcome, correlation analyses were performed separately in pregnant and non-pregnant patients (Figure 4). Among the six retained genera, significant associations were confined to stromal p16-positive cells in the pregnant group. Stromal p16 positivity was negatively correlated with *Lactobacillus* abundance (Spearman ρ = −0.485, *p* = 0.0049, q = 0.0295) and positively correlated with *Delftia* abundance (ρ = 0.421, *p* = 0.0165, q = 0.0494) (Figure 5). Neither association was significant in the non-pregnant group. No significant implantation outcome-specific associations were observed for luminal epithelial p16-positive cells after correction for multiple testing.

### 3.6. Species-Level Associations Within the Lactobacillus Genus

**Figure 6:**
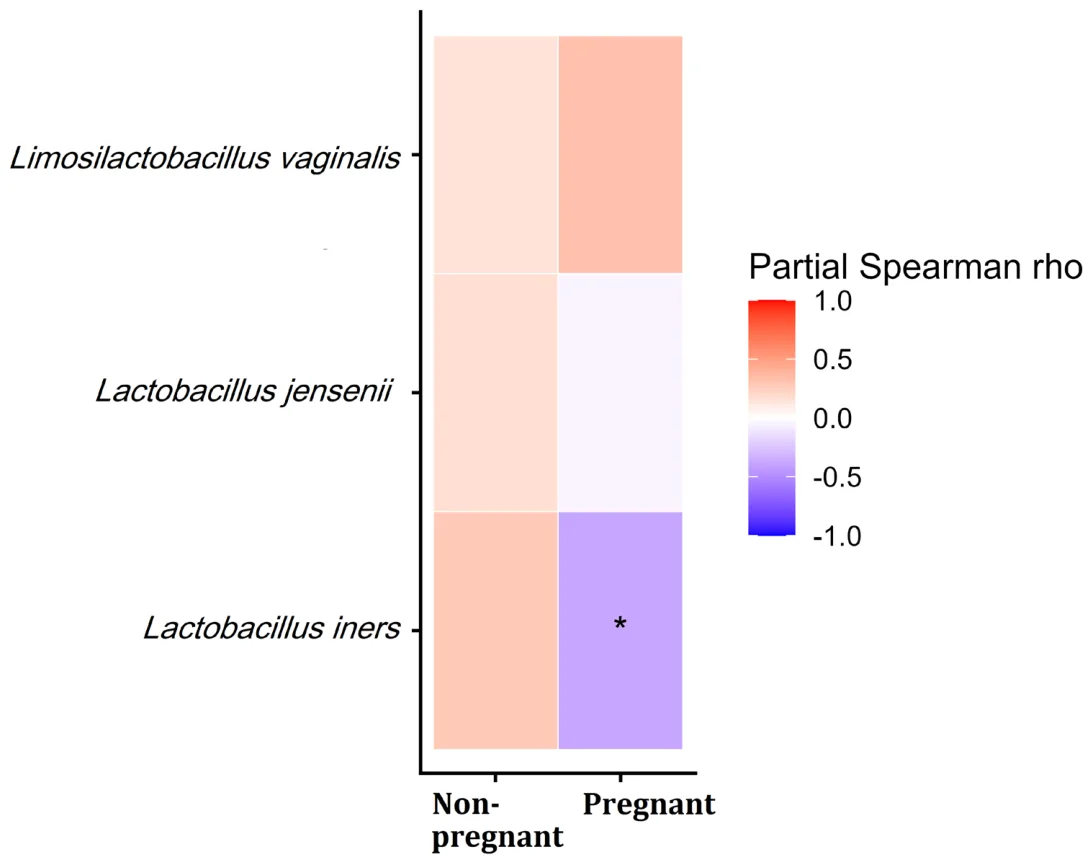
Species-level associations between stromal p16-positive cells and *Lactobacillus* species according to implantation outcome. Heatmap showing partial Spearman correlation coefficients between stromal p16-positive cell abundance and the relative abundance of the three bacterial species detected in at least 20% of endometrial samples. Correlations are presented separately for non-pregnant and pregnant patients. Color intensity reflects the strength and direction of the association, with positive correlations shown in red and negative correlations in blue. * indicates *p* < 0.05.

Species-level analyses were restricted to bacterial species detected in at least 20% of endometrial samples in order to ensure sufficient prevalence for statistical evaluation. Analysis of the three most prevalent species in the study cohort (*Lactobacillus iners, Limosilactobacillus vaginalis* (formerly *Lactobacillus vaginalis*), and *Lactobacillus jensenii*) revealed distinct associations with stromal p16 expression (Figure 6). Among the examined species, *Lactobacillus iners* demonstrated the strongest relationship with stromal senescence. In pregnant patients, stromal p16 abundance was negatively associated with *Lactobacillus iners* (ρ = −0.38, *p* = 0.03), whereas no significant association was observed in non-pregnant patients (ρ = 0.28, *p* > 0.05). *Limosilactobacillus vaginalis* showed a positive correlation coefficient with stromal p16 expression in pregnant women (ρ = 0.32, *p* < 0.10), but this association did not reach statistical significance. *Lactobacillus jensenii* was not significantly associated with stromal p16 expression in either implantation group. These findings suggest that senescence-associated microbial alterations occur at the species level and may not be fully captured by genus-level analyses.

### 3.7. Differences in Bacterial Abundance According to Stromal p16 Expression

**Figure 7:**
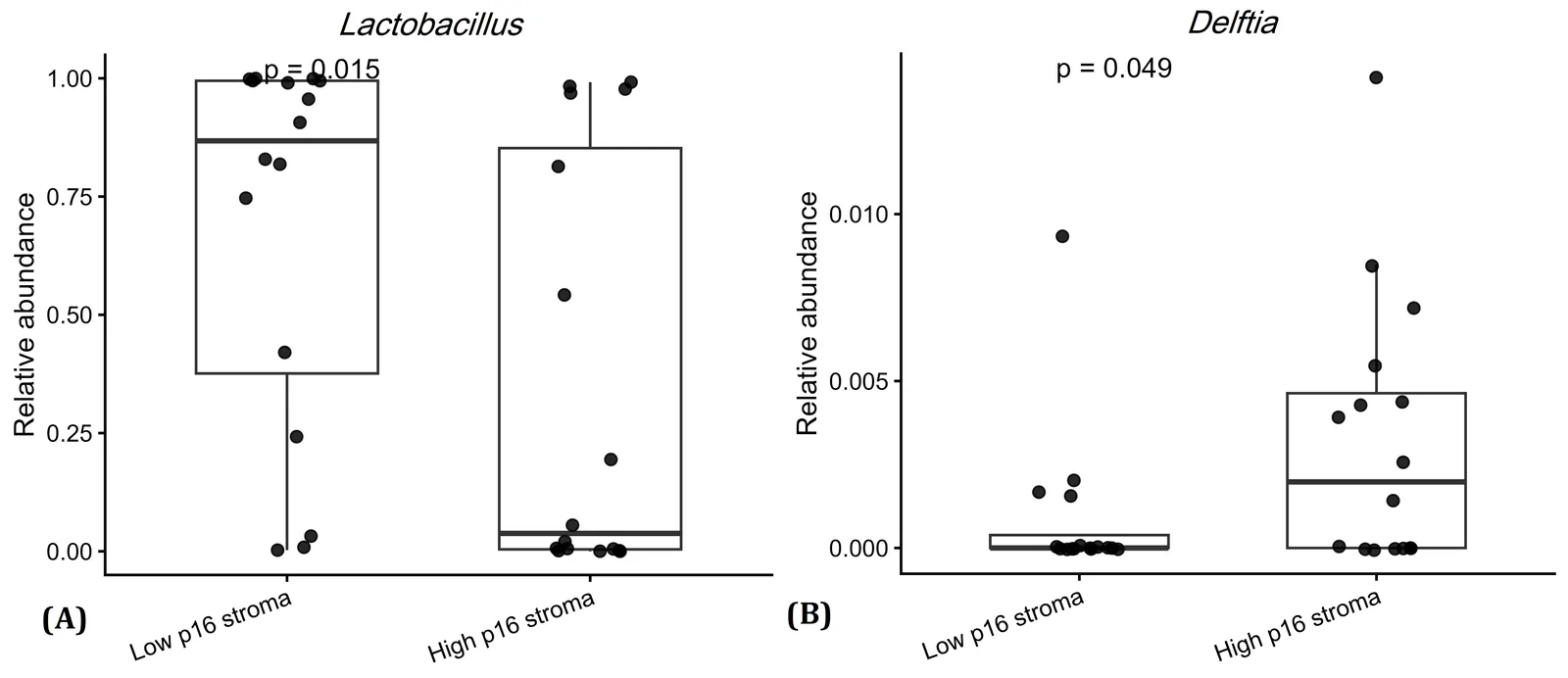
Relative abundance of *Lactobacillus* and *Delftia* according to stromal p16 expression. (**A**) Relative abundance of *Lactobacillus* in samples with low and high stromal p16 expression. (**B**) Relative abundance of *Delftia* in samples with low and high stromal p16 expression. Samples were stratified according to stromal p16 abundance using the predefined threshold. Boxes represent the interquartile range (IQR), horizontal lines indicate the median, whiskers represent 1.5 × IQR, and dots correspond to individual samples. Group comparisons were performed using the Mann–Whitney U test. *p*-values are shown within each panel.

For exploratory analyses, samples were stratified into low and high stromal p16 groups using the cohort median stromal p16 value as the cutoff (0.63% p16-positive stromal cells). Samples with stromal p16 values ≤ 0.63% were classified as low stromal p16, whereas samples with values > 0.63% were classified as high stromal p16. Although substantial inter-individual variability in bacterial relative abundance was observed, samples with high stromal p16 expression exhibited significantly lower relative abundance of *Lactobacillus* (*p* = 0.015) and significantly higher relative abundance of *Delftia* (*p* = 0.049) compared with samples characterized by low stromal p16 abundance (Figure 7). These findings were consistent with the correlation analyses and further supported an association between stromal senescence and microbial composition.

### 3.8. Stromal p16-Associated Microbial Interaction Networks

**Figure 8:**
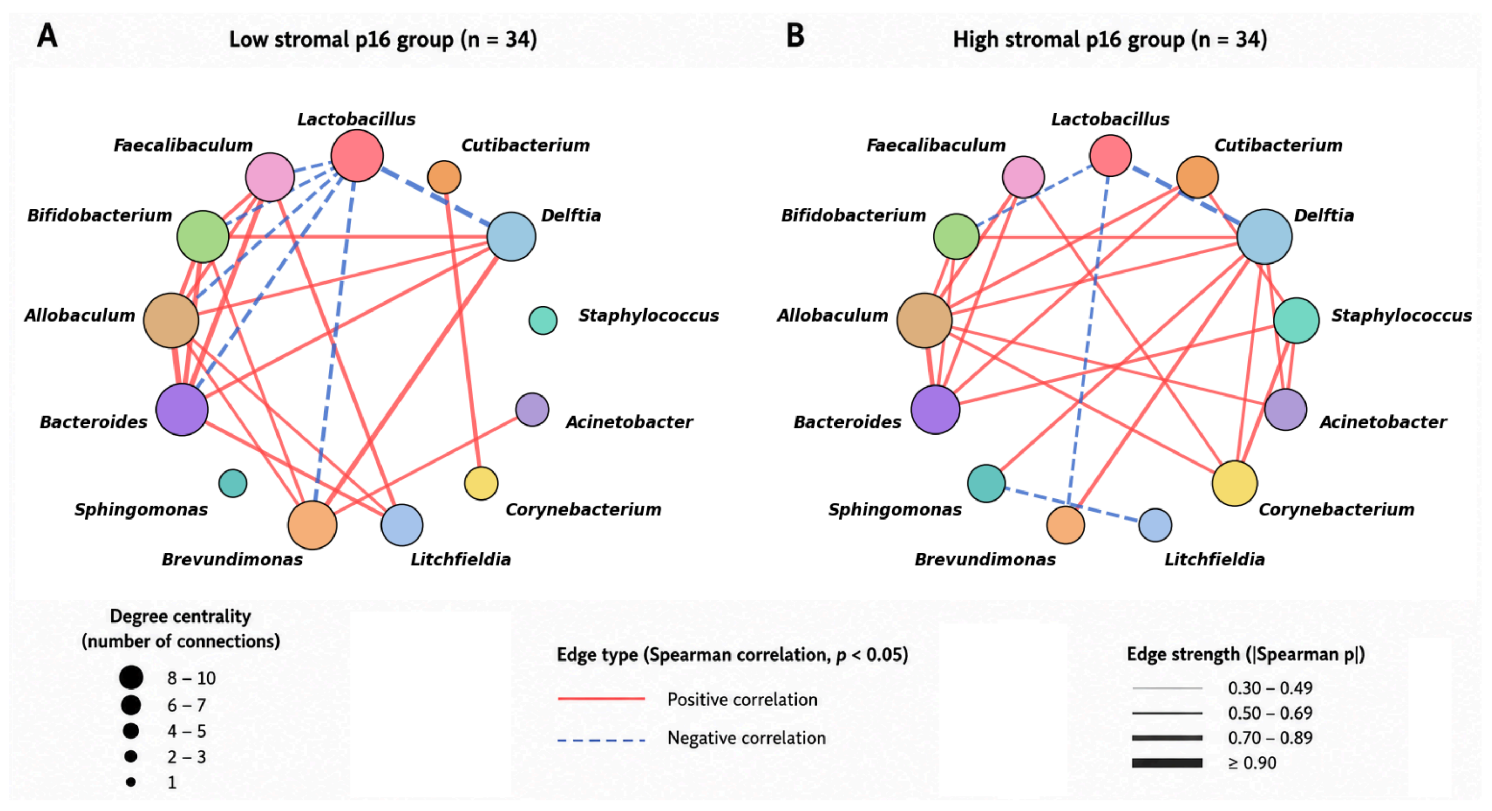
Endometrial microbial co-occurrence networks according to stromal p16 expression. (**A**) Co-occurrence network in samples with low stromal p16 expression. (**B**) Co-occurrence network in samples with high stromal p16 expression. Networks were constructed using 13 bacterial genera detected in at least 10% of the study samples. Nodes represent bacterial genera, with node size proportional to degree centrality. Edges represent significant pairwise Spearman correlations (*p* < 0.05); positive and negative associations are indicated by solid and dashed lines, respectively. Edge width reflects the absolute strength of the correlation coefficient. Identical node positions are used in both panels to facilitate direct comparison of network organization between the two groups.

To investigate whether stromal senescence was associated with alterations in microbial community organization, genus-level co-occurrence networks were constructed separately for the low- and high-stromal p16 groups using 13 bacterial genera detected in at least 10% of the study samples. The two networks showed a similar overall number of significant interactions, with 23 edges in the low-p16 group and 24 edges in the high-p16 group (Figure 8). Positive associations predominated in both networks but were more frequent in the high-p16 group (20 vs. 17 positive edges), whereas negative associations were more frequent in the low-p16 group (6 vs. 4 negative edges).

Network density was slightly higher in the high-p16 group (0.308 vs. 0.295), which was also reflected by a modestly higher average degree (3.69 vs. 3.54). In contrast, the low-p16 network exhibited a higher clustering coefficient (0.485 vs. 0.408) and a higher mean absolute correlation strength (|ρ| = 0.521 vs. 0.429). These findings indicate that stromal p16 status was associated primarily with differences in the organization and strength of microbial interactions rather than with a marked expansion or contraction of the overall co-occurrence network.

Network centrality analysis further revealed differences in the taxa occupying highly connected positions. In the low-p16 network, *Allobaculum* showed the highest degree, followed by *Lactobacillus*, *Bacteroides*, and *Bifidobacterium*. In the high-p16 network, *Delftia* and *Allobaculum* displayed the highest degree centrality. Together, these changes suggest a redistribution of microbial interaction architecture according to stromal p16 expression.

**Figure A1:**
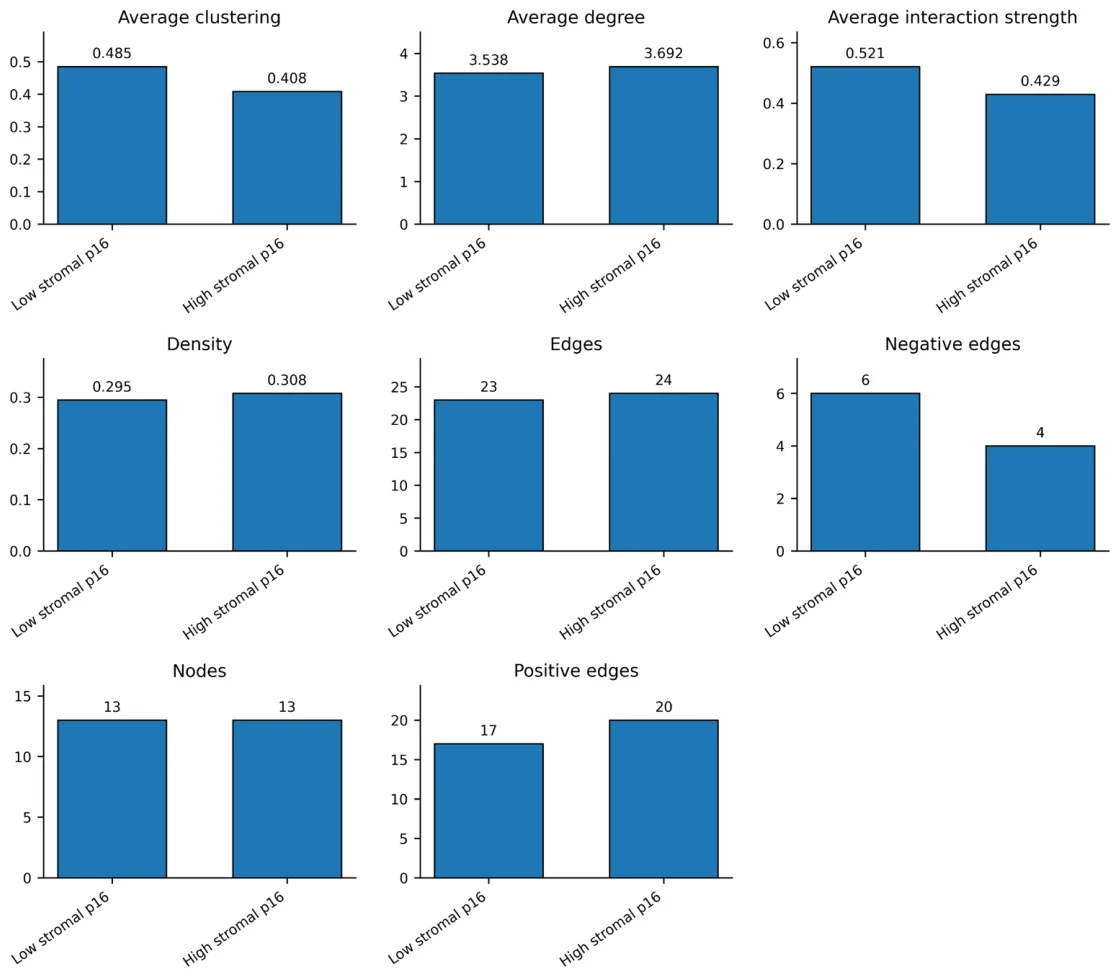
Global topological characteristics of endometrial microbial co-occurrence networks according to stromal p16 expression. Comparison of network metrics between samples with low and high stromal p16 abundance. Displayed parameters include clustering coefficient, average degree, average interaction strength (mean absolute Spearman correlation coefficient), network density, number of edges, number of nodes, and numbers of positive and negative associations. Networks were constructed using significant genus-level Spearman correlations (*p* < 0.05) among the 13 bacterial genera retained for exploratory network analysis.

Overall, stromal p16 expression was associated with reorganization of microbial interaction patterns, including differences in network clustering, interaction strength, and the identity of highly connected taxa (Figure A1).

### 3.9. Associations Between Stromal p16 Expression and Microbial Taxa According to Implantation Outcome

Stratified correlation analysis identified implantation outcome-specific patterns in the associations between stromal p16 expression and the six bacterial genera retained after abundance- and prevalence-based filtering. In the pregnant group, stromal p16 expression was negatively correlated with *Lactobacillus* abundance (Spearman ρ = −0.485, *p* = 0.0049, q = 0.0295) and positively correlated with *Delftia* abundance (ρ = 0.421, *p* = 0.0165, q = 0.0494), whereas neither association was significant in the non-pregnant group (ρ = −0.041, *p* = 0.812 and ρ = 0.137, *p* = 0.424, respectively).

**Figure 9:**
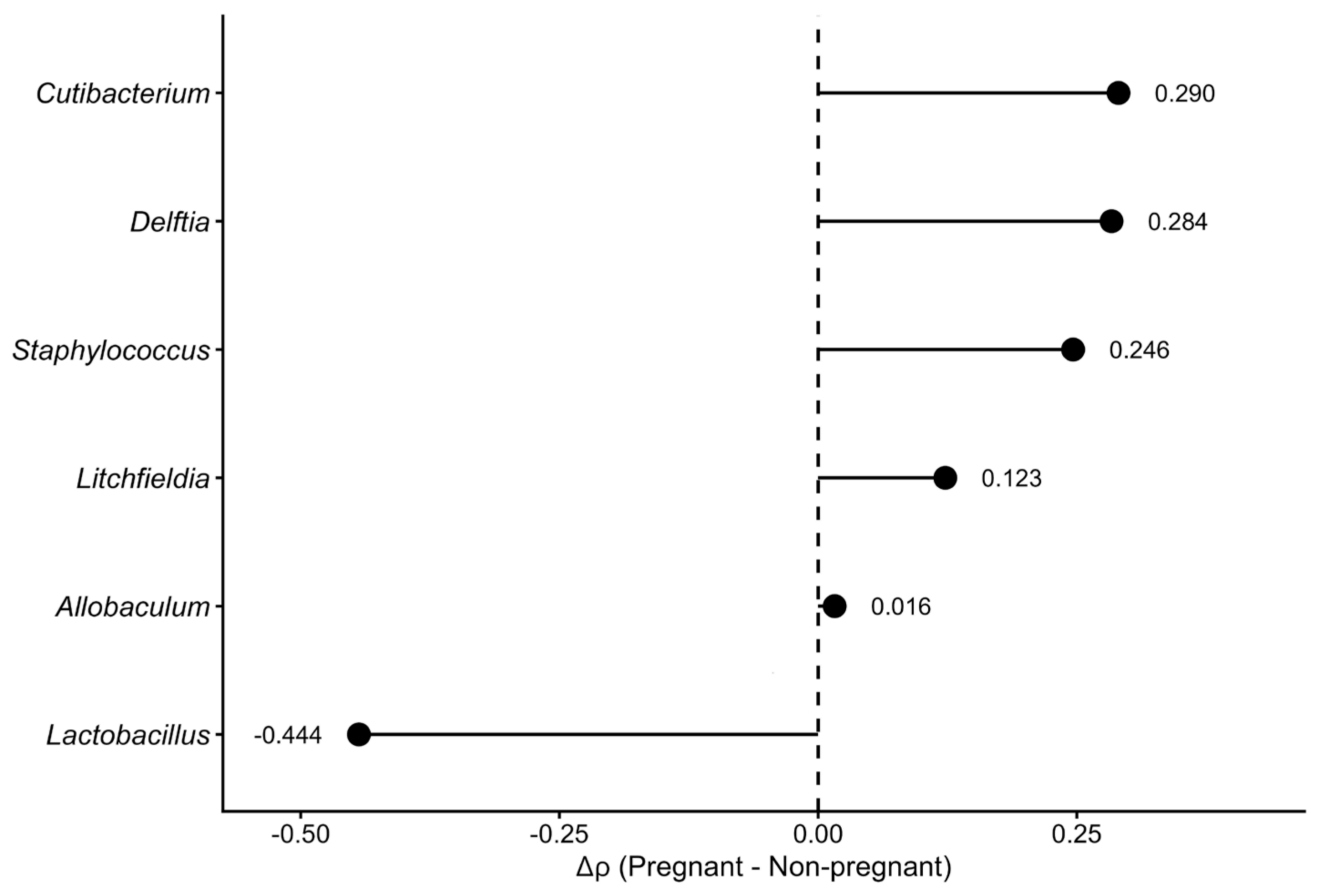
Differential correlations between stromal p16-positive cells and endometrial bacterial genera according to implantation outcome. Values represent the difference in Spearman correlation coefficients (Δρ = ρPregnant − ρNon-pregnant) for associations between stromal p16-positive cells and the six bacterial genera retained after abundance- and prevalence-based filtering. Positive Δρ values indicate stronger positive or weaker negative associations in the pregnant group, whereas negative values indicate stronger negative or weaker positive associations in the pregnant group. The vertical dashed line indicates no between-group difference (Δρ = 0). Between-group differences in correlation coefficients were evaluated using Fisher’s r-to-z transformation with Benjamini–Hochberg correction for multiple testing. None of the differential correlations remained statistically significant after FDR correction.

Differential correlation analysis was subsequently used to formally compare the strength of these associations between implantation groups (Figure 9). The largest between-group difference was observed for *Lactobacillus* (Δρ = −0.444), reflecting a stronger negative association in the pregnant group, followed by *Cutibacterium* (Δρ = 0.290), *Delftia* (Δρ = 0.284), and *Staphylococcus* (Δρ = 0.246). However, none of the between-group differences reached statistical significance after multiple-testing correction (all q > 0.05). Thus, although the stratified analyses identified significant correlations of stromal p16 with *Lactobacillus* and *Delftia* specifically within the pregnant group, formal comparison did not provide statistically significant evidence that the corresponding correlation coefficients differed between implantation groups.

## 4. Discussion

The present study investigated the relationship between endometrial cellular senescence and the endometrial microbiota during the window of implantation. While previous studies have independently linked both endometrial senescence and microbial dysbiosis to adverse reproductive outcomes, the interaction between these two biological processes remains largely unexplored. To our knowledge, this is the first study to investigate associations between stromal p16-positive endometrial cells and endometrial microbiota composition during the window of implantation.

Our analysis demonstrated that stromal p16 positivity was associated with distinct alterations in the endometrial microbiota. Higher stromal p16 expression was accompanied by reduced relative abundance of *Lactobacillus* and increased abundance of *Delftia*. These associations were observed across complementary analytical approaches, including overall correlation analyses, direct comparisons between high- and low-senescence groups, and implantation-stratified analyses. The recurrence of these findings across complementary analyses supports a potential biological relationship between endometrial senescence and microbial composition, although confirmation in larger independent cohorts is warranted. Our findings support the hypothesis that stromal senescence may represent one component of a bidirectional host–microbiota regulatory axis within the receptive endometrium. One plausible biological basis for this hypothesis is the ability of senescent stromal cells to actively remodel their surrounding microenvironment through the senescence-associated secretory phenotype (SASP).

Cellular senescence is characterized by irreversible cell-cycle arrest and the acquisition of a senescence-associated secretory phenotype (SASP), which includes numerous pro-inflammatory cytokines, chemokines, growth factors, and matrix-remodeling enzymes [20,21,42]. Through these secreted mediators, senescent cells can profoundly influence the surrounding tissue microenvironment, modulate immune cell recruitment, and alter intercellular communication. In the endometrium, transient and tightly regulated senescence has been proposed as a physiological component of decidualization and tissue remodeling during the implantation window [22,23,24]. However, excessive accumulation or persistence of senescent cells may disrupt endometrial homeostasis, promote chronic inflammation, impair decidual function, and contribute to reproductive failure [19,22,23,43]. These observations support the concept that the balance between physiological and pathological senescence may represent an important determinant of endometrial receptivity.

Interestingly, several women in the non-pregnant group exhibited relatively high p16-positive luminal epithelial cell abundance despite the absence of significant differences at the group level. Although increased epithelial p16 expression may be induced by inflammatory or other cellular stress-related stimuli, all women with active genital tract infection and histologically confirmed chronic endometritis were excluded from the study. Therefore, these observations may reflect biological heterogeneity within the non-pregnant cohort rather than clinically evident inflammatory pathology. Nevertheless, the biological significance of elevated epithelial p16 expression remains uncertain and warrants further investigation using complementary inflammatory markers, additional senescence-associated biomarkers, and functional assessment of the senescence-associated secretory phenotype (SASP).

Previous studies from our group have demonstrated associations between endometrial senescence, local immune cell populations, plasma cell infiltration, and reproductive outcomes [25,26,27,35]. These observations are consistent with growing evidence that senescent cells actively participate in shaping local immune responses through SASP-mediated signaling and interactions with both innate and adaptive immune cells [44,45]. In parallel, accumulating data indicate that microbial communities can influence tissue immune homeostasis and inflammatory signaling, while immune-mediated mechanisms contribute to the maintenance of microbial community structure [10,46]. The present study extends this framework by identifying a previously unexplored association between stromal p16-senescence and the endometrial microbiota. Together, these findings support the concept that cellular senescence may represent a central component of a broader endometrial regulatory network integrating immune, microbial, and tissue-remodeling processes.

An important observation of the present study was that the strongest senescence–microbiota associations were detected among women who subsequently achieved successful implantation. In particular, stromal p16 expression was negatively associated with *Lactobacillus* and positively associated with *Delftia* in the pregnant group, whereas neither association reached statistical significance in the non-pregnant group. However, formal differential correlation analysis did not demonstrate statistically significant between-group differences in correlation coefficients after correction for multiple testing. Therefore, these findings should not be interpreted as evidence of implantation-specific effects, but rather as exploratory evidence that senescence–microbiota relationships may vary according to reproductive context. Greater biological homogeneity within the pregnant subgroup may also have contributed to the more readily detectable associations. These observations require confirmation in larger independent cohorts with sufficient statistical power for formal between-group comparisons.

Following application of the predefined abundance- and prevalence-based filtering criteria, *Lactobacillus* and *Delftia* emerged as the genera showing the most prominent associations with stromal p16 expression. Both genera were therefore examined in greater detail because of the consistency of their associations across complementary analyses and their potential relevance to endometrial microbial ecology and reproductive outcomes.

*Lactobacillus* remains the most extensively studied component of the female reproductive tract microbiota. Numerous studies have associated *Lactobacillus*-dominant microbial communities with favorable reproductive outcomes, including improved implantation rates and pregnancy success, although results remain inconsistent across cohorts, sequencing approaches, and definitions of microbial dominance [5,6,47,48,49]. In the present study, *Lactobacillus* abundance was inversely related to stromal p16 expression, although the overall association did not remain statistically significant after FDR correction. Furthermore, among women with low stromal senescence, higher *Lactobacillus* abundance was associated with successful implantation, whereas this relationship disappeared in the high-senescence subgroup. These findings indicate a close association between stromal p16 expression and *Lactobacillus* abundance. One possible explanation is that senescence-associated inflammatory signaling may modify host–microbial interactions, thereby diminishing the capacity of *Lactobacillus*-dominated communities to support endometrial homeostasis and receptivity. Alternatively, reduced *Lactobacillus* abundance may contribute to a microenvironment that favors chronic inflammatory processes and the accumulation of senescent cells. Although causality cannot be established from the present data, the observed association supports a close relationship between *Lactobacillus* abundance and endometrial tissue integrity during the implantation window.

An additional observation was the consistent association between *Delftia* and stromal p16 positivity. Samples with high stromal p16 expression demonstrated significantly higher relative abundance of *Delftia*, and positive correlations between *Delftia* abundance and stromal p16 levels were observed. Notably, this finding is consistent with our previous prospective study evaluating the endometrial microbiota and implantation outcome following transfer of a single euploid embryo, in which *Delftia* was significantly enriched among women who failed to achieve pregnancy [8]. Similar observations have been reported in studies investigating the reproductive tract microbiota, where the presence of *Delftia* has been associated with non-*Lactobacillus*-dominant microbial communities and less favorable reproductive outcomes [50]. The recurrence of this association across complementary analyses and study objectives suggests that *Delftia* may warrant further investigation as a potential marker of an altered endometrial microenvironment. However, given the recognized challenges of contamination in low-biomass microbiota studies and the limited biological evidence regarding the role of Delftia in the human endometrium, these findings should be considered exploratory until confirmed in independent cohorts using complementary sequencing approaches.

Despite these observations, the biological role of *Delftia* within the human endometrium remains poorly understood. Whether *Delftia* directly contributes to local inflammatory signaling or instead reflects broader ecological changes within the endometrial microbiota remains unknown. Nevertheless, its repeated association with both implantation failure and increased stromal p16 expression highlights this genus as a potentially informative marker of endometrial dysfunction and a candidate for future mechanistic investigations.

At the species level, the three prevalent bacterial species detected in at least 20% of samples were *Lactobacillus* species, emphasizing the dominant contribution of this genus to the endometrial ecosystem. Correlation analyses revealed species-specific association patterns. *Lactobacillus iners* was positively correlated with stromal p16 expression in non-pregnant women and negatively correlated in pregnant women, with the latter association reaching statistical significance. This observation suggests that this relationship may depend on the reproductive context. In contrast, *Limosilactobacillus vaginalis* showed a positive correlation coefficient with stromal p16 cells in pregnant women, but the association was not statistically significant. *Lactobacillus jensenii* demonstrated no significant associations with stromal p16 expression in either group. These observations are consistent with increasing evidence that individual *Lactobacillus* species differ substantially in their biological properties and interactions with the host [51]. In particular, *L. iners* occupies a unique position within the female reproductive tract microbiota, as it has been associated with both healthy and transitional microbial states and may exhibit context-dependent effects on reproductive health [52,53,54]. The opposing associations observed for *L. iners* according to implantation outcome raise the possibility that species-specific host–microbiota interactions may influence the relationship between microbial composition and endometrial senescence. Although these findings should be interpreted cautiously due to the relatively small sample size, they support the concept that individual Lactobacillus species should not be considered functionally equivalent when evaluating the reproductive tract microbiota.

Network-based analyses further demonstrated that stromal p16 expression was associated not only with changes in individual taxa but also with alterations in microbial community organization. Using the same predefined set of bacterial genera in both groups, the high-p16 network contained a slightly greater number of significant associations and showed modestly higher network density and average degree, whereas the low-p16 network exhibited a higher clustering coefficient and greater mean interaction strength. The balance between positive and negative associations also differed, with positive correlations being more frequent in the high-p16 network. These findings suggest that stromal p16 expression may be associated with reorganization of microbial interaction patterns rather than simply with expansion or contraction of the microbial network.

Microbial co-occurrence network analyses provide information that extends beyond conventional abundance-based approaches by characterizing community organization, ecological interactions, hub taxa, and network robustness [55,56,57,58]. Changes in network topology may reflect shifts in community stability, resilience, and functional organization that are not detectable through differential abundance analyses alone. Interestingly, the observed differences between low- and high-senescence networks suggest that cellular senescence may be associated with broader ecological restructuring of the endometrial microbial community. Although co-occurrence networks do not establish direct biological interactions, the observed differences in network topology and interaction patterns support the hypothesis that stromal senescence may be linked to altered microbial community organization within the endometrium. Collectively, these findings suggest that stromal senescence may be associated not only with shifts in individual bacterial taxa but also with broader reorganization of microbial community structure within the endometrium.

Several mechanisms may explain the observed associations. Senescent stromal cells produce a wide range of inflammatory mediators, growth factors, proteases, and extracellular matrix-modifying enzymes collectively known as the senescence-associated secretory phenotype (SASP), which can substantially alter tissue homeostasis [20,44]. Through these mediators, senescent cells may influence epithelial barrier integrity, nutrient availability, local immune surveillance, and antimicrobial activity, thereby creating conditions that favor specific microbial communities. In addition, SASP-associated cytokines, including IL-6, IL-8, and other pro-inflammatory mediators, have been implicated in the establishment of chronic low-grade inflammatory environments capable of reshaping host–microbiota interactions [19,42].

Conversely, microbial products may contribute to the development of cellular senescence through persistent inflammatory stimulation, oxidative stress, DNA damage, and activation of innate immune pathways [58,59]. Increasing evidence from studies in other tissues suggests that microbiota alterations may contribute to aging-associated inflammatory processes, while age-related changes in host physiology can reciprocally influence microbial community structure [60]. Therefore, the relationship between senescence and microbiota composition is likely bidirectional, with each process potentially influencing and amplifying the other. Collectively, these biologically plausible mechanisms provide a conceptual framework for interpreting the context-dependent associations observed in the present study and generate testable hypotheses for future mechanistic investigations.

The present study has several strengths. All samples were collected during the window of implantation, implantation outcome was assessed following transfer of a single euploid embryo, and both microbial and senescence-related analyses were performed on the same endometrial specimens. These design features reduced major sources of biological variability and allowed direct investigation of host–microbiota interactions.

Several limitations should also be acknowledged. The study cohort was relatively small, limiting statistical power for analyses of low-abundance taxa. To reduce the influence of sparse and very low-abundance taxa, genus-level correlation analyses were restricted using predefined abundance- and prevalence-based criteria; however, this conservative filtering approach may have excluded biologically relevant but less prevalent microorganisms. The cross-sectional design precludes conclusions regarding causality between microbial alterations and senescence. In addition, stratification according to implantation outcome further reduced the sample size available for subgroup analyses. Consequently, subgroup-specific associations should be interpreted as exploratory and require confirmation in larger independent cohorts. Moreover, although several correlations reached significance within the pregnant subgroup, formal comparisons of correlation coefficients between implantation groups did not remain significant after correction for multiple testing. Similarly, the microbial co-occurrence network analyses were exploratory and descriptive in nature. Although differences in network architecture were observed between groups, formal statistical comparison of network topology was beyond the scope of the present study and should be addressed in future investigations using dedicated network comparison approaches. Furthermore, embryo transfer was performed several months after endometrial biopsy. Although all biopsies and subsequent embryo transfers were performed under the same standardized hormonal protocol and at the same stage of endometrial preparation (progesterone day 5), temporal biological variation in the endometrial microbiota and local tissue microenvironment between cycles cannot be completely excluded. Therefore, the observed associations should be interpreted as reflecting relationships between baseline endometrial characteristics and subsequent implantation outcome rather than the endometrial state at the time of embryo transfer. Although p16^INK4a^ is one of the most widely used markers of cellular senescence, it does not fully capture the complexity of the senescent phenotype and may also be expressed under certain non-senescent biological conditions. Therefore, the use of additional senescence-associated markers and functional assessment of the senescence-associated secretory phenotype (SASP) would provide a more comprehensive characterization of endometrial senescence in future studies. In addition, although strict sampling procedures were applied, contamination remains a recognized challenge in low-biomass microbiota studies. To minimize this risk, negative extraction controls and phosphate-buffered saline (PBS) controls processed through the entire analytical workflow were included during sequencing; however, the possibility of low-level background contamination cannot be completely excluded. Finally, 16S rRNA sequencing provides limited functional information and cannot determine the biological activity of individual microorganisms. Furthermore, although 16S rRNA gene sequencing enables robust characterization of bacterial community composition, species-level taxonomic assignment remains limited for certain closely related taxa, particularly within the order Lactobacillales. Therefore, the observed species-level associations should be interpreted with caution and require confirmation using higher-resolution approaches such as shotgun metagenomic sequencing.

## 5. Conclusions

The present study suggests that stromal, but not luminal epithelial, p16-positive senescent cells are associated with distinct alterations in the endometrial microbiota composition during the window of implantation. Increased stromal p16 positivity was associated with lower *Lactobacillus* abundance and higher *Delftia* abundance, while species-level analyses revealed differential associations involving prevalent *Lactobacillus* species.

Importantly, the strongest senescence–microbiota associations were observed in women who subsequently achieved successful implantation. Although these subgroup-specific findings require confirmation in larger independent cohorts, they raise the possibility that coordinated relationships between stromal senescence and microbial communities may represent a previously underrecognized feature of the receptive endometrial microenvironment. In addition to changes in individual taxa, stromal p16 expression was associated with differences in microbial interaction networks, indicating broader effects on microbial community organization.

Together, these findings support the concept that endometrial senescence and the microbiota are interconnected biological processes during the implantation window. The observed associations highlight a potential link between cellular senescence, microbial community structure, and endometrial function. Further prospective studies incorporating predefined, shorter biopsy-to-transfer intervals and, where feasible, repeated sampling are required to determine the directionality, temporal stability, and potential clinical relevance of these relationships.

## Figures and Tables

**Table 1:** Baseline demographic, reproductive, and treatment characteristics of the study population (n = 68) according to implantation outcome.

Characteristic	Non-Pregnant (*n* = 36)	Pregnant (*n* = 32)	*p*-Value
Age (years)	35.00 [32.00–39.00]	35.50 [32.00–39.25]	0.912
BMI (kg/m^2^)	23.25 [22.30–26.07]	24.20 [21.83–28.05]	0.796
Previous IVF cycles, *n*	2.00 [2.00–3.00]	2.00 [1.75–2.25]	0.238
Previous embryo transfers, *n*	3.00 [2.00–4.00]	2.00 [2.00–3.25]	0.510
Endometrial thickness at ET (mm)	9.10 [8.25–10.03]	9.45 [8.80–10.38]	0.212
Time between biopsy and embryo transfer (months)	4.00 [3.00–5.00]	3.50 [3.00–6.00]	0.796
Primary infertility diagnosis:			0.615
Male factor	9 (25.0%)	10 (31.2%)	
Tubal factor	4 (11.1%)	5 (15.6%)	
Ovulatory factor	8 (22.2%)	4 (12.5%)	
Unexplained infertility	7 (19.4%)	9 (28.1%)	
Mixed factors	8 (22.2%)	4 (12.5%)	

Abbreviations: BMI, body mass index; IVF, in vitro fertilization; ET, embryo transfer. Continuous variables are presented as median [interquartile range]. Comparisons between groups were performed using the Mann–Whitney U test. Categorical variables are presented as *n* (%) and were compared using Fisher’s exact test or χ^2^ test, as appropriate.

## Data Availability

The original data presented in the study are openly available in zenodo.org at DOI: https://doi.org/10.5281/zenodo.20758364.

## Supplementary Materials

The following supporting information can be downloaded at https://www.mdpi.com/article/10.3390/microorganisms14091868/s1: Table S1: Sample-level sequencing statistics and relative abundances of bacterial genera evaluated for downstream filtering and analyses. The dataset presents, for each endometrial sample, the number of raw paired-end reads, reads retained after quality filtering and chimera removal, the number of observed amplicon sequence variants (ASVs), and the relative abundances of the bacterial genera included in the downstream analyses. Relative abundance values are expressed as proportions of the total genus-level abundance within each sample.

## Abbreviations

The following abbreviations are used in this manuscript:

ASV	Amplicon Sequence Variant
BMI	Body Mass Index
DNA	Deoxyribonucleic Acid
ET	Embryo Transfer
FDR	False Discovery Rate
hCG	human chorionic gonadotropin
HRT	Hormone Replacement Treatment
IHC	Immunohistochemistry
IQR	Interquartile Range
IVF	In Vitro Fertilization
NGS	Next-Generation Sequencing
OTU	Operational Taxonomic Unit
PBS	Phosphate-Buffered Saline
PCR	Polymerase Chain Reaction
PGT-A	Preimplantation Genetic Testing for Aneuploidy
QIIME	Quantitative Insights Into Microbial Ecology
rRNA	Ribosomal Ribonucleic Acid
SASP	Senescence-Associated Secretory Phenotype
SD	Standard Deviation
SILVA	Silva Ribosomal RNA Database
ρ	Spearman Correlation Coefficient
Δρ	Difference in Spearman Correlation Coefficients

## Appendix A

**Table A1:** Summary of genera containing candidate contaminant ASVs identified by prevalence-based screening with negative extraction controls.

Genus	Total ASVs Assigned to Genus	Candidate Contaminant ASVs (n)	Retained for Primary Genus-Level Analysis	Retained for Network Analysis	Differentially Abundant Between Groups	Contributed to Major Biological Conclusions
*Acinetobacter*	40	3	No	Yes	No	No
*Sphingomonas*	57	2	No	Yes	No	No
*Cutibacterium*	38	1	Yes	Yes	No	No
*Faecalibacterium*	22	1	No	Yes	No	No
*Methylobacterium*	20	1	No	No	No	No
*Brevundimonas*	18	1	No	Yes	No	No
*Unclassified Micrococcaceae*	11	1	No	No	No	No
*Tepidimonas*	6	1	No	No	No	No
*Bradyrhizobium*	4	1	No	No	No	No
*Serratia*	2	1	No	No	No	No
*Sphingopyxis*	1	1	No	No	No	No

Candidate contaminant ASVs were identified using the prevalence method implemented in the decontam package and subsequently mapped to their corresponding bacterial genera. The table summarizes whether genera containing candidate contaminant ASVs were retained for the primary genus-level association analyses or for the exploratory network analysis. Genera retained for the primary genus-level analyses met the predefined abundance- and prevalence-based filtering criteria, whereas genera included in the exploratory network analysis were selected using the predefined prevalence-based criterion. For most genera, candidate contaminant ASVs represented only a subset of all ASVs assigned to the corresponding genus; however, this proportion varied among genera. Therefore, identification of a candidate contaminant ASV was interpreted at the feature level and was not considered sufficient to classify all sequences assigned to the corresponding genus as contaminants. None of the candidate contaminant genera corresponded to the principal taxa underlying the main biological conclusions of the study.
